# Correction to ‘Latent Profile Analysis of Moral Sensitivity and Influencing Factors Among Nursing Students in China’

**DOI:** 10.1002/nop2.70810

**Published:** 2026-09-24

Tian, Weiyan , Xianjuan Gou , Mingdan Li , Lingjun Luo , Peng Luo , and Mei Chen . 2025. “Latent Profile Analysis of Moral Sensitivity and Influencing Factors Among Nursing Students in China.” Nursing Open 12, no. 12: e70373. 10.1002/nop2.70373.PMC1266274641315003

This paper was originally published as a ‘Discursive Paper’ Article Type, but has now been corrected to an ‘Empirical Research Quantitative’.

We apologize for this error.

